# Classification of Depressive and Schizophrenic Episodes Using Night-Time Motor Activity Signal

**DOI:** 10.3390/healthcare10071256

**Published:** 2022-07-05

**Authors:** Julieta G. Rodríguez-Ruiz, Carlos E. Galván-Tejada, Huizilopoztli Luna-García, Hamurabi Gamboa-Rosales, José M. Celaya-Padilla, José G. Arceo-Olague, Jorge I. Galván Tejada

**Affiliations:** 1 Unidad Académica de Ingeniería Eléctrica, Universidad Autónoma de Zacatecas, Jardín Juarez 147, Centro, Zacatecas 98000, Mexico; jr.ruiz68@uaz.edu.mx (J.G.R.-R.); hlugar@uaz.edu.mx (H.L.-G.); hamurabigr@uaz.edu.mx (H.G.-R.); arceojg@uaz.edu.mx (J.G.A.-O.); gatejo@uaz.edu.mx (J.I.G.T.); 2 CONACYT, Universidad Autónoma de Zacatecas, Jardín Juarez 147, Centro, Zacatecas 98000, Mexico; jose.celaya@uaz.edu.mx

**Keywords:** depression, schizophrenia, machine learning, random forest, night-time

## Abstract

Major depressive disorder (MDD) is the most recurrent mental illness globally, affecting approximately 5% of adults. Furthermore, according to the National Institute of Mental Health (NIMH) of the U.S., calculating an actual schizophrenia prevalence rate is challenging because of this illness’s underdiagnosis. Still, most current global metrics hover between 0.33% and 0.75%. Machine-learning scientists use data from diverse sources to analyze, classify, or predict to improve the psychiatric attention, diagnosis, and treatment of MDD, schizophrenia, and other psychiatric conditions. Motor activity data are gaining popularity in mental illness diagnosis assistance because they are a cost-effective and noninvasive method. In the knowledge discovery in databases (KDD) framework, a model to classify depressive and schizophrenic patients from healthy controls is constructed using accelerometer data. Taking advantage of the multiple sleep disorders caused by mental disorders, the main objective is to increase the model’s accuracy by employing only data from night-time activity. To compare the classification between the stages of the day and improve the accuracy of the classification, the total activity signal was cut into hourly time lapses and then grouped into subdatasets depending on the phases of the day: morning (06:00–11:59), afternoon (12:00–17:59), evening (18:00–23:59), and night (00:00–05:59). Random forest classifier (RFC) is the algorithm proposed for multiclass classification, and it uses accuracy, recall, precision, the Matthews correlation coefficient, and F1 score to measure its efficiency. The best model was night-featured data and RFC, with 98% accuracy for the classification of three classes. The effectiveness of this experiment leads to less monitoring time for patients, reducing stress and anxiety, producing more efficient models, using wearables, and increasing the amount of data.

## 1. Introduction

Depression is the fourth disease causing disability worldwide, and in the U.S is the second most prevalent illness, followed by hypertension [1,2]. Furthermore, the COVID-19 pandemic has aggravated this situation. Catherine K. Ettman et al. reported that depressive feelings increased from 8.5% to 27.8% during the period in quarantine [3]. This phenomenon could render depression the most prevalent disease causing serious expenses for countries’ healthcare and severe health consequences for the population. Major depressive disorder (MDD) is the most severe form of depression. To obtain an MDD diagnosis, patients may present symptoms such as hopelessness, melancholy, sadness, low energy, sleep disturbance, low appetite, and interference to ordinary activities such as work, study, or home tasks for at least two weeks [4,5]. On the other hand, psychotic disorders have a prevalence of 3.89 per 1000, representing a relatively low prevalence of illness [6]. However, schizophrenia is estimated to be the fifth cause of lost years among men, and the sixth for women. Schizophrenia is a complex mental disorder distinguished by producing delusion or hallucinations in patients [7]. It can qualify as negative, with depressive symptoms, or positive, accompanied by aggressive behavior [8]. Schizophrenic patients present alterations in the prefrontal and parietal–temporal cortex, altering their verbal, memory, functional, and processing speed [9]. Depression and schizophrenia commonly share symptoms, as several cases of schizophrenic patients suffer from depression simultaneously, and unipolar depressive patients tend to deploy psychotic episodes [10]. The worst symptom of depression and schizophrenia can be suicidal thoughts and attempts, and suicide. Around 700,000 people die every year because of suicide from depression, and for schizophrenic patients, the rate may increase by 12 times [11,12,13]. Almost 80% of schizophrenic patients suffer some activity disruption while sleeping or during the day [14]. In addition, the prevalence of insomnia in adult patients with depressive disorder increases to 75% or 90% if we count all the sleep disturbances [15,16]. In the process of mental illness diagnosis, the Diagnostic and Statistical Manual of Mental Disorders (5th edition) is the most common in occidental countries. However, the scientific and medical community has questioned the relevance of this manual limited to describing mental disorders, relapsing the diagnosis in the experience of the medical care specialist, and its criteria [17]. New techniques to reach precision in psychiatry have become the primary goal. In the psychiatry field, the lack of measurement and patient monitorization is notable, and to solve it, precision psychiatry has appeared [18]. Precision psychiatry involves distinct domains to improve the diagnosis, prognosis, treatment, classification of illness, and the characterization and measurement of mental disorders by applying techniques using neuroimaging, machine learning, deep learning, and others [18,19]. For example, to analyze psychiatric behavior, scientists use speech analysis, activity monitorization, facial expression recognition, heart rate, medical-note analysis, and even social media publications [20]. Machine learning is the perfect combination of statistics and computer science, making it a promising tool to decipher the complex information obtained in psychiatric patients [21]. However, studies on mental illness multiclass classification are scarce, and there are even fewer using motor activity signals to identify different mental disorders. Hugo G. Schnack et al. used magnetic resonance images (MRIs) to extract features of gray matter densities, and classified schizophrenia–healthy controls, bipolar disorder–schizophrenia, and bipolar disorder–healthy controls using support vector machine models, with accuracy results of 90%, 88%, and 59%, respectively, demonstrating the efficiency of MRI and machine learning in identifying schizophrenic and bipolar disorder patients [22]. Wei Han et al. proposed supervised convex non-negative matrix factorization to create a connectivity pattern map from resting-state functional magnetic resonance imaging (rs-fMRI) in patients with schizophrenia. Using brain network connectivity maps from 21 patients with schizophrenia and 25 patients with MDD, and a support vector machine classifier, they accomplished an accuracy classification of 82.6% [23]. Other MRI approaches included rest–activity rhythm data to correlate the anatomy of brain patients and their physical behavior in daily activities [24,25]. The circadian rhythm in psychiatric patients is crucial in diagnosis and treatment. Nevertheless, psychiatrists rarely use techniques or devices to obtain reliable data about the patient’s rest–activity rhythm [26]. Therefore, the addition of physical activity monitoring is essential for psychiatric evaluation. The systematic review of Yuuki Tazawa et al. has confirmed the effectiveness of using actigraphy devices to compare patients with mood disorder activity against healthy controls. They used statistics such as p-value and standard deviation to demonstrate significant differences in activity levels, particularly during night-time, wake after sleep, and morning wake [27]. Regarding schizophrenia and actigraphy data, the systematic review of Zi Ying Wee et al. showed differences in activity levels between schizophrenic patients and nonpsychotic disorders, and even differences in activity patterns related to schizophrenia severity [28]. Further, the correlation between activity data and psychopathology is evident and can help medical practice in improving the patient status and reintegration to every-day activities [28]. Other approaches are monitoring activity levels while using antidepressant or antipsychotic medications, which can improve treatment and avoid lethal secondary effects [29]. Similarly, in a statistical analysis of activity signals in healthy controls, and depressive and schizophrenic patients, the results suggested that the activity of patients with schizophrenia tends to be more structured and with lower energy levels, even compared with depressive ones [26]. Nevertheless, multiclass models to classify more than two psychiatric disorders are sparse; commonly, classification is led with data from patients with one type of disorder and healthy controls. Fewer models for multiclass classification in mental illness, such as the ones mentioned above, employed data from MRIs, and compared with motor activity from a wearable, it is a less invasive method, cheaper, and reduces stress in patients. Therefore, the contribution of this paper is the multiclass classification of schizophrenic, depressive, and healthy-control motor activity with an analysis of the different stages of the day, following the premise that, during the night, alterations in the physical movement of this type of patient are more evident. In previous work, classifying depressive episodes using activity signals improved performance using only night activity counts [30]. One of the most important symptoms to avoid in patients with schizophrenia is an alteration of the sleep–wake cycle because of the implications on everyday activities; additionally, it is the best lapse to validate the efficacy of drugs used in this type of patient [31]. In addition, suicidal ideation is highly associated with sleep–wakefulness and other sleep disturbances, which makes it essential to look more into night-time episode research [32]. The final objective of a data-mining process is following the knowledge discovery in databases (KDD) framework, which establishes well-defined and concrete steps to obtain valuable information from data, and it has been adequate for knowledge discovery in healthcare [33]. The article is structured following the steps of the KDD framework. First, Section 2 describes every step of extracting knowledge from data and assembling a model: selection, preprocessing, transformation, data mining, and evaluation. Then, Section 3 reports the evaluation outcomes of each model, and Section 4 describes the contribution in detail.

## 2. Materials and Methods

**Figure 1:**
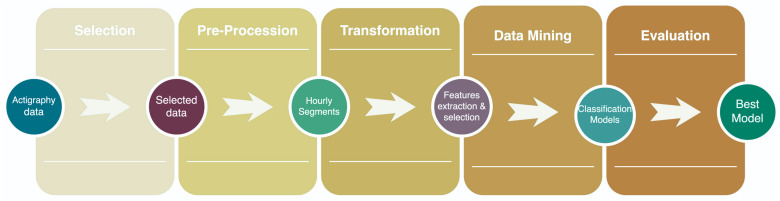
Data-mining process used in this paper to classify depressive, schizophrenic, and healthy-control episodes.

Machine learning (ML) is an abstraction of the feature data used to train it, as this is the source of learning to predict or classify new samples in the future regarding supervised algorithms [34]. Otherwise, an unsupervised model automatically makes a classification or prediction using patterns in data without needing a training phase [35]. Given that machine learning is a set of techniques currently used in the data-mining process to obtain knowledge from data or databases, few methodologies or frameworks have been developed to encourage good practices in data mining. Knowledge discovery from databases (KDD) is a data-mining process with well-established stages: pre-KDD, selection, preprocessing, transformation, data mining, interpretation/evaluation, and post-KDD [36]. Figure 1 shows the main stages of the KDD process that led to obtaining the best model for multiclass classification. It is an iterative and nonsequential process allowing for much flexibility to obtain valuable information from data. In pre-KDD, the analysis of the pertinence and importance of the project is conducted taking into account the final user point of view, and post-KDD knowledge provides the final model description report. Table 1 briefly describes the activities produced in every stage of this work. In related works seen in the literature can be found similar steps to obtain classification models for psychiatric disorders, with a higher tendency in binary classification. For example, Zhuozheng Wang et al. used electroencephalography (EGG) and a 2D convolutional network for depression diagnosis and achieved accuracy of 92% [37]. In a multiclass classification of major depressive disorder, bipolar disorder, schizophrenia, and generalized anxiety disorder, Caroline Wanderley Espinola et al. reached 75.27% accuracy using a random forest classifier with 300 trees [38]. Another relevant work using the same activity data used in this paper was published by Rohit Kumar Bondugula et al., who achieved 86.60% accuracy in a bidirectional recurrent neural network (BRNN) for the binary classification of schizophrenic patients and healthy controls [39].

**Table 1:** KDD steps and activities involved in this work.

KDD Process	
Pre-KDD	Precision psychiatry using ML algorithms’ principal objectives of treatment response analysis, early identification, suicide prevention, real-time monitoring, and subclassified actual mental disorders [33]. In addition, ML models avoid generic diagnoses, providing new classifications of individuals by their features [40].
Selection	The Depresjon and Psykose datasets contain monitor-activity counts of patients with depression and schizophrenia, respectively.
Preprocessing	All patients’ activity count data are concatenated into a single matrix, standardized, transposed, and grouped by hours.
Transformation	After hourly segmentation, data are grouped into subsets following the day stage: morning (06:00–11:59), afternoon (12:00–17:59), evening (18:00–23:59), and night (00:00–05:59).
Data Mining	Classification of depressive, schizophrenic, and control episodes is performed with a random forest classifier.
Interpretation/evaluation	Precision, recall, F1 score, MCC, and accuracy measure every model’s effectiveness to identify healthy, schizophrenic, and depressive episodes concerning the day stage.
Post-KDD	It is not limited to this written report.

### 2.1. Selection

Clinical actigraphy use began in the 1990s. These sensors became popular, and their use in psychiatry quickly brought new implications and directions to the field [28]. The Depresjon and Psykose datasets contain motor activity data sensed using Actiwatch, Cambridge Neurotechnology Ltd, England, model AW4 on the right wrist of every patient and control [41,42]. This actigraph contains a piezoelectric sensor to measure acceleration, tension, or force. Measurement occurs when an external factor applies pressure on the piezoelectric mass, and this force generates an electrical response [43]. The AW4 Actiwatch has a frequency sampling of 32 Hz and a sensitivity of 0.05 g, and its configuration counts the voltage produced by the device every minute. The final activity count per minute is proportional to the intensity of the movement. The Psykose dataset contains activity data from 22 patients with a schizophrenia diagnosis and 32 healthy controls: one comma-separated values (CSV) file for each participant with the timestamp, date, and activity count column [41]. With the same structure, the Depresjon dataset contains CSV files with the data of 23 depressive patients and the same 32 healthy controls [42]. In addition, both datasets include patient information such as age, gender, days of monitorization, treatment, and specifications around the mental disorder. The severity of the symptoms caused by depression was measured using the Montgomery–Asberg depression rating scale (MADRS). Five of the depressive patients were hospitalized during the data extraction [42]. For measuring the psychopathology of schizophrenic patients, researchers used the Brief Psychiatric Rating Scale (BPRS) [41]. Enrique Garcia-Ceja et al. collected and published the Depresjon dataset for free use with the experimental results. In the same way, Petter Jakobsen et al. published the Psykose dataset with a fully opened license for research and educational purposes [41,42].

**Figure 2:**
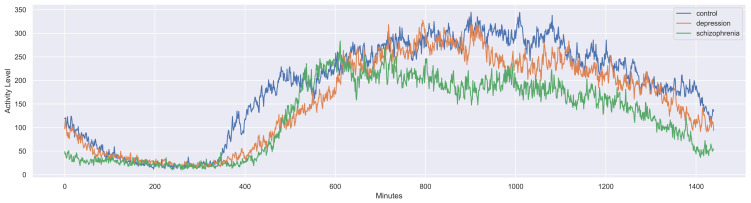
Plot of the mean activity level during all monitored days from every type: schizophrenic patients, depressive patients, and healthy controls. It starts at 00:00, and every point corresponds to one of the 1440 min in one day.

Every CSV contained an array of activity levels for approximately 13 days. Figure 2 shows the mean activity level for every minute during the 24 h of the day, starting at 00:00 for healthy, depressive, and schizophrenic patients.

This plot shows differences in the three subjects’ activity levels. During the entire day, activity levels perdure differently, with the schizophrenia signal being the one with lower activity levels, and depression being similar to the healthy-control signal but with less intensity.

### 2.2. Preprocessing

Once all files from every class had been grouped into one dataset, the next step was to normalize or standardize the data. Machine-learning data standardization is essential to obtain better results with ML algorithms. Depending on the nature of the data, the method to normalize was selected; in this case, standardization with standard deviation was the best option to avoid the increment of noise using other methods such as min–max normalization. Equation (2) is the Zscore formula, where *x* is every activity count, mu is the mean of all activity counts of the class, and sigma is its standard deviation.

(1) $$Zscore=\frac{x-\mu }{\sigma }$$

This process converts the original activity signal into, Zscore, improving the comparability among the data and converting the mean to zero. After standardization, all data were concatenated into one matrix with timestamp, time, and activity level columns, and 2,106,734 rows. This matrix data were transposed and segmented into hourly lapses to obtain comparable instances. In this process, the matrix then contained rows of 60 activity counts corresponding to a specific hour of the day. Lastly, the hours were grouped by day stage: night, morning, afternoon, and evening.

### 2.3. Transformation

Data segments needed to be transformed by signal characterization following two main processes, feature extraction and feature selection. Feature extraction focuses on obtaining significant features that effectively describe the original signal. It can be related to signal domain transformation, statistical or central tendency metric extraction, dimensionality reduction, and other pattern-searching techniques. On the other hand, feature selection directly implicates dimension reduction by selecting only the most meaningful features through different methods, such as a predictive model that looks for the best feature combinations to produce the classification [44].

#### 2.3.1. Feature Extraction

The main goal in this phase is feature extraction from every hourly lapse of the activity signal. In related works, Swapnil Sayan Saha et al. used an accelerometer signal for human action recognition (HAR), and demonstrated that extracting quantitative and statistical features achieved better fall detection and HAR with 97% and 93% accuracy, respectively [45]. Qi Wei Oung et al. also focused their research on human movement in the healthcare field for the more specific detection of freezing of gait (FoG), a severe symptom of Parkinson’s patients. Their research concentrated on extracting mathematical features from the time and frequency domains using the signal of a three-axis accelerometer and obtaining a model with 99.96% accuracy, classifying FoG [46]. On the basis of these feature extraction methods and their results, this work is focused on extracting central tendency measures and other statistical moments in the time domain. From this procedure, 23 features per hour were obtained, reducing the 60 activity original counts to only 23 features describing the activity levels every hour. Every feature description by its equation is shown in Table 2.

#### 2.3.2. Feature Selection

This process focuses on reducing noisy features from the feature space, increasing the accuracy of the model, improving the algorithm’s performance, and improving data storage for future applications [47]. The most used methods to eject feature selection are correlation analysis, supervised and unsupervised classification algorithms, univariate analysis, recursive feature elimination, forward elimination, feature importance, principal component analysis, and genetic algorithms [48].

In this work, recursive feature elimination with cross-validation (RFE-CV) and random forest classifier (RFC) selected the number of features and their best combination. First, REF-CV recursively trained an RFC algorithm with all the dataset’s original features and calculated the model’s accuracy through testing its classification. This process was repeatd until the last feature, and in every loop, features negatively affecting the model’s accuracy were eliminated. Table 3 shows the set of best features per day-stage dataset.

### 2.4. Data Mining

KDD’s data mining process step focuses on selecting the best machine-learning algorithm, testing the models, and obtaining classification results. The first step is choosing the algorithm, and on the basis of documentation, RFC implementations achieved high accuracy in movement classification, mental-illness machine-learning diagnosis, and activity recognition [49,50]. The random forest classifier (RFC) is an ensemble machine-learning algorithm employing regression and decision trees for classifications. It characterizes by producing decision tree classifiers with subsamples of the original dataset to increment the accuracy of the model at the same time while avoiding overfitting [51]. RFC configuration for the classification of schizophrenic, depressive, and control episodes was:

- 900 trees in the forest;
- at least three samples were required to split a node;
- to be at a leaf node, the minimum required six samples;
- the maximal number of leaf nodes in a tree was 90;
- not bootstrapping the samples used the entire dataset to construct every tree.

These hyperparameters were selected from a grid search with a cross-validation method. However, the grids had been calculated with a previous random search to know the best ranges for every parameter. RFC is a supervised model, which means that it needs to learn from the data before producing classifications with it. Table 4 shows the instances used for training and testing every subset of data according to the day stage. In addition to the typical evaluation of 70–30 for training and testing the model, cross-validation was implemented fivefold. It is helpful in corroborating how accurate the model is and how it would perform in practice.

K-fold cross-validation splits the data into *k* subsets or folds. In this process, the ML model trains with k−1 folds and then evaluates the performance with the remaining folds. Cross-validation (CV) repeats this process for all the *k* folds with a different subset of data for testing.

**Figure 3:**
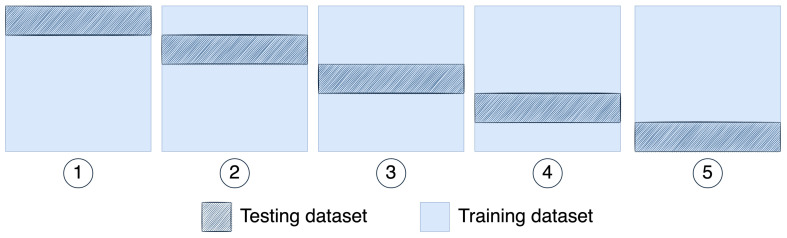
The fivefold cross-validation uses resampling data to create five different datasets to evaluate the model’s performance. Sets 1 to 5 proportions are 80% of the data for training the model and the rest 20% for testing it.

Figure 3 shows the process for fivefold cross-validation; it was implemented for every day-stage model. In other words, the data were divided into a random 80–20%,and the model was evaluated with five different testing subsets.

### 2.5. Evaluation

There are four primary metrics to evaluate the performance of a model to classify classes: true positive (TP), true negative (TN), false positive (FP), and false negative (FN). These metrics are commonly used in binary classifications, but the same metrics can be calculated by summarizing the values corresponding to the same columns or rows in multiclass classification. These metrics also calculate precision, recall, F1 score, and accuracy.

(2) $$precision=\frac{TP}{TP+FP}$$

Precision Equation (2) means the proportion of correct positive predictions, that is, how many predictions are correct from a specific class.

(3) $$recall=\frac{TP}{TP+FN}$$

Recall in Equation (3) calculates the proportion of actual correctly identified positives, that is, from all the true samples, how many are correct as of the specific class.

(4) $$F1score=2\frac{\left(precision)(recall\right)}{precision+recall}$$

F1score Equation (4) combines the recall and precision metrics in a harmonic mean, and calculates the classification’s general efficiency.

(5) $$MCC=\frac{TN*TP-FN*FP}{\sqrt{\left(TP+FP)(TP+FN)(TN+FP)(TN+FN\right)}}$$

Matthews Correlation Coefficient (MCC) calculates the association between the predicted classification and the actual data; its value range was from −1 to 1, where −1 is noncorrelation, and 1 indicates total correlation. MCC in Equation (5) summarizes the results exposed on a confusion matrix and uses the four basic metrics regarding the model precision. In this case, MCC was calculated using multiclass classification, taking one class as positive and the rest as negative, and then summarizing the results of every class to obtain a single value per model.

(6) $$Accuracy=\frac{TP+TN}{TP+TN+FP+FN}$$

Lastly, accuracy in Equation (6) obtains the fraction of correct predictions. It is used to evaluate the cross-validation technique with multiclass classification.

## 3. Results

Every RFC model was trained with 70% of the data, and the remaining 30% were used to test its performance.

**Figure 4:**
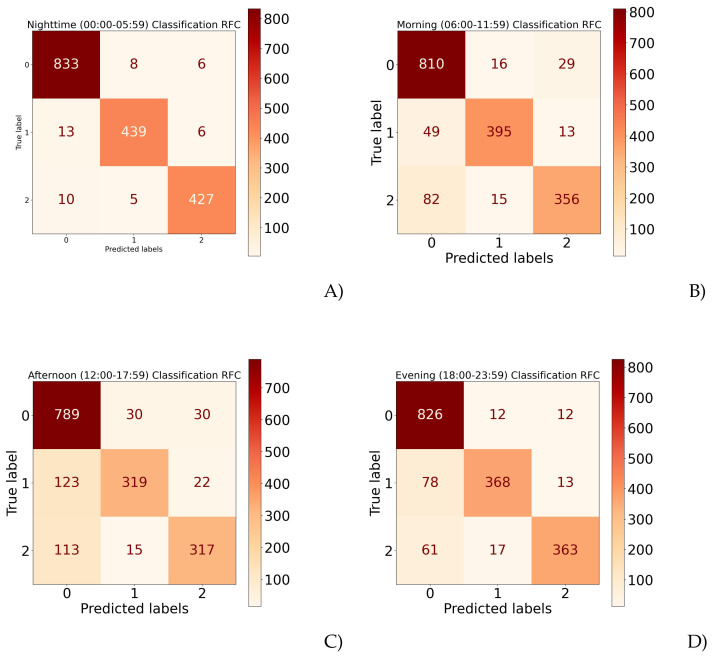
Confusion matrices by time segment. (**A**) Night-time; (**B**) morning; (**C**) afternoon; (**D**) evening. Note: 0 means healthy control, 1 depressive, and 2 schizophrenic episodes.

To visualize the results obtained from every model classification, a confusion matrix was calculated for every model. Figure 4A shows the proportion of correct classifications for each class using activity data from 00:00 to 05:59.

Figure 4B–D show the classification performance for the rest of the data separated by day stage: 06:00–11:59, 12:00–17:59, and 18:00–23:59, respectively.

There was a data imbalance for every class in the confusion matrices, as shown in Figure 4. There were more samples of healthy controls than schizophrenic and depressive ones; for that reason, the MCC metric was considered to measure how well the model classified despite the imbalanced data.

For every day-stage model, fivefold CV was performed following the previous description; from this process, the maximal, minimal, and overall accuracy of the classification was obtained. Table 5 shows these accuracies per model.

Additionally, precision, recall, F1 score, and MCC provided more detail about the model performance during classification instead of focusing only on accuracy. Table 6 shows the results for every metric, class, and model. In multiclass classification, the specific evaluation per class helps in examining how well the model classifies one class against the others.

**Figure 5:**
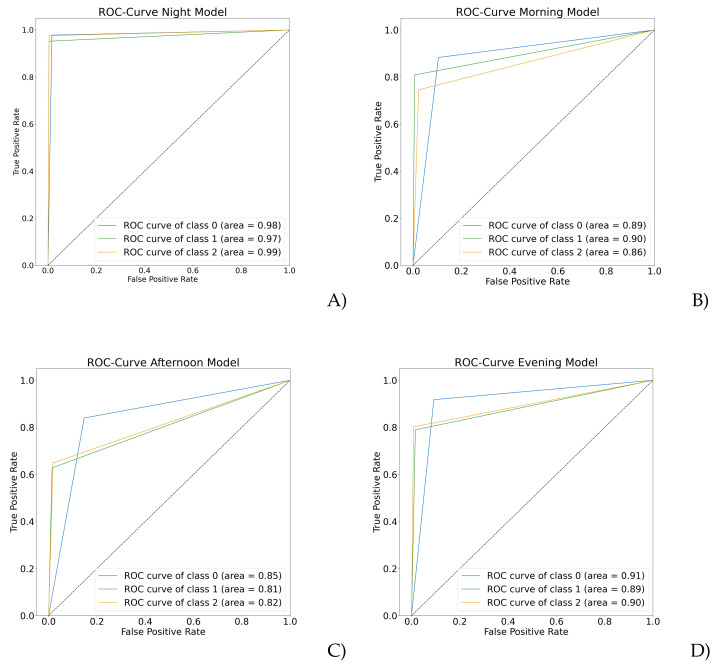
ROC curve plots by time segment. (**A**) Night-time; (**B**) morning; (**C**) afternoon; (**D**) evening. Note: 0 means healthy control, 1 depressive, and 2 schizophrenic episodes.

Moreover, the ROC curve was calculated for every time segment model. The ROC curve plots the true-positive rate against the false-positive rate and measures, and gives a general model representation to diagnose. Since this is a multiclass classification, one technique is needed to calculate the ROC curve for every class, such as a binary classification. Predictions were calculated using “one vs. rest”; in this way, the evaluated class was positive, while the rest meant negative. Figure 5 shows the ROC curve for each model. The blue line means healthy control, green is depression, and orange is the capability for classifying schizophrenia.

The experimentation in this article was executed using Python version 3.8.8. The main tools used were Pandas for data manipulation and early stages of the data-mining process, and scikit-learn for the machine-learning stage and the evaluation of the models. The hardware used was an Apple M1 processor with 16 GB RAM. The most exhaustive process for computational resources was the grid search to define the best hyperparameters for the RFC model using all data; it took around 56 min to finish all the random combinations.

## 4. Discussion and Conclusions

The multiclass classification of mental illness using ML is sparse; commonly, models perform binary classification between disease and healthy controls. The main idea of this work was to assemble an efficient model to classify more than one mental disorder against healthy controls using motor activity and RFC. This paper proposed an RFC model for thee multiclass classification of schizophrenia, depression, and healthy controls using night-time activity level data with accuracy of 98%. The experimental results proved the model’s effectiveness in identifying episodes of depression and schizophrenia, and healthy controls using RFC against previous related work where more computationally expensive algorithms were used, for instance, CNN and BRNN, with a significant increase in accuracy. Previous works such as Han W. et al. model used magnetic resonance imaging to classify depression and schizophrenia, achieving 82.6% accuracy [23]. In comparison, the model proposed in this paper improves the classification accuracy and uses a less invasive method to acquire the data, namely, a bracelet with an accelerometer. In addition, another multiclass model using MRI, presented by Schnack et al., achieved 90% accuracy in identifying bipolar disorder, schizophrenia, and healthy controls using gray matter features. Activity data collection using wearables is one of the best cost-effective and noninvasive options. The effectiveness of monitoring night-time activity in mental health for its correlation to sleep disorders and daily retardation provides more meaningful information compared to the entire day [30]. In addition, this specific model can assist in diagnosis, analyze treatment effectiveness and the circadian rhythm, and even help patients’ primary caregivers. Nightt-ime monitoring in psychiatric patients regards the improvement of their lifestyle by observing the evolution of treatment, identifying sleep disturbances, or recognizing the disease using only this specific lapse of data. According to Berle et al., the motor signal of depressive and schizophrenic patients shows a statistical difference during the night, agreeing with these results. Therefore, further data analysis with schizophrenic and depressive data, and even relating it with the treatments can clarify and improve outcomes [26]. The process described in this paper to obtain the best model can be helpful in other types of signal data analysis, standardization, and statistical feature extraction on the time domain, and selecting the best features can improve the classification of models. The standardization step is especially essential for ML algorithms, as it provides the same importance or influence to all the data features, and helps the model in converging more easily and being computationally faster. In synergy with the standardization, RFC achieved high accuracy for the night-time model, and the rest of the models obtained 80% or more correct classifications. From the confusion matrices and ROC curve plots, it can be determined that healthy control activity patterns are more easily classified. However, data classes for every model training and evaluation are not balanced; data from healthy controls represent at least 50% of the total data classes, but in this experimentation, any resampling method could have been used. Future work implementing techniques to balance data may increase the accuracy and allow for the model to correctly classify all classes in the same terms. Feature extraction following the methodology of Qi Wei Oung et al. could develop a transformation to the frequency domain, and obtain features in both the time and frequency domains to improve the accuracies of classification [46]. Following the ROC curve plots, the green line representing depression against control and schizophrenic data remained with the lowest values, except in the morning model with 0.90 of the area. In this model, the best classification against the others was depression, which may inquire about specific activity patterns of retardation during wake-up and the starting of activities around depression patients. In addition, this model can provide information about sleep disorders or sleeping-pill treatment efficacy. For the rest of the models, it was easier to detect schizophrenic than depressive episodes. Most schizophrenic patients are hospitalized for an average of 24 years, which affects their daily routines. However, for specialists, it could be interesting to notice data patterns concerning the morning and afternoon models. Since it is more challenging to differentiate schizophrenic from control and depressive episodes, it may be related to circadian rhythm alteration. The datasets used in this paper did not have records of activities per patient. In the future, this kind of data could provide more information about day stages and render the segmentation more accurate in identifying specific patterns while, for example, sleeping, taking treatment pills, and sleeping during the day. Lastly, the hyperparameters used in this experimentation could be taken to classify other data types, evaluate the algorithm’s configuration, and verify that they are optimal in another kind of problem.

## Figures and Tables

**Table 2:** Features extracted from hourly segments of motor activity signals.

Name	Equation
Mean	$\bar{x}=\frac{1}{n}\sum_{i=1}^{n}x_{i}$
Sum	$sum=\sum_{i=1}^{n}x_{i}$
Maximum	Maximal value
Minimum	Minimal value
Median	$\tilde{x}=\frac{\left(n+1\right)}{2}$
Standard deviation	$S_{x}=\sqrt{\frac{\sum_{i=1}^{n}{\left(x_{i}-\bar{x}\right)}^{2}}{n-1}}$
First decile	$D_{1}=value\;\;of\left[\frac{n+1}{10}\right]th\;\;data$
Second decile	$D_{2}=value\;\;of\left[\frac{2(n+1)}{10}\right]th\;\;data$
First quantile	$Q_{1}=\frac{1}{4}\left(n+1\right)thterm$
Third decile	$D_{3}=value\;\;of\left[\frac{3(n+1)}{10}\right]th\;\;data$
Fourth decile	$D_{4}=value\;\;of\left[\frac{4(n+1)}{10}\right]th\;\;data$
Second quantile	$Q_{2}=\frac{2}{4}{\left(n+1\right)}^{th}term$
Sixth decile	$D_{6}=value\;\;of\left[\frac{6(n+1)}{10}\right]th\;\;data$
Seventh decile	$D_{7}=value\;\;of\left[\frac{7(n+1)}{10}\right]th\;\;data$
Third quantile	$Q_{3}=\frac{3}{4}{\left(n+1\right)}^{th}term$
Eighth decile	$D_{8}=value\;\;of\left[\frac{8(n+1)}{10}\right]th\;\;data$
Ninth decile	$D_{9}=value\;\;of\left[\frac{9(n+1)}{10}\right]th\;data$
Kurtosis	$K=\frac{1}{n}\frac{\sum_{i=1}^{n}{\left(x_{i}-\bar{x}\right)}^{4}}{S_{x}^{4}}$
Mean absolute deviation	$MAD=\frac{1}{n}\sum_{i=1}^{n}\left|x_{i}-\bar{x}\right|$
Standard error of mean	$SE_{\bar{x}}=\frac{S_{x}}{\sqrt{n}}$
Skewness	$S=\frac{1}{n}\frac{\sum_{i=1}^{n}{\left(x_{i}-\bar{x}\right)}^{3}}{S_{x}^{3}}$
Variance	$s^{2}=\frac{\sum_{i=1}^{n}{\left(x_{i}-\bar{x}\right)}^{2}}{n-1}$
Unique	distinct elements count
where *n* is the size of sample, $x_{i}$ is an item of the sample, $S_{x}^{4}$ is the fourth standardized moment, and $S_{x}^{3}$ is the third standardized moment.	

**Table 3:** Motor activity level proportion per class.

Day Stage	No. Features	Features
00:00–05:59	5	min, quantile10, quantile20, quantile25, quantile30
06:00–11:59	7	min, median, quantile10, quantile20, quantile25, quantile30, quantile40
12:00–17:59	8	max, min, quantile10, quantile20, quantile25, quantile30, quantile40, quantile60
18:00–23:59	6	min, quantile10, quantile20, quantile25, quantile30, quantile40

**Table 4:** Motor activity level proportion per class.

Day Stage	Training Instances	Testing Instances	Features
00:00–06:00	6116	2622	5
06:00–12:00	5051	2165	6
12:00–18:00	4809	2061	8
18:00–00:00	4761	2041	7

**Table 5:** Fivefold cross-validation results using accuracy as the evaluation metric.

Model	Accuracy	
Nighttime (00:00–05:59)	Maximum	98.62%
	Minimum	97.25%
	Overall	98.24%
Morning (06:00–11:59)	Maximum	88.44%
	Minimum	87.47%
	Overall	87.97%
Afternoon (12:00–17:59)	Maximum	81.63%
	Minimum	80.27%
	Overall	80.92%
Evening (18:00–23:59)	Maximum	91.26%
	Minimum	88.97%
	Overall	89.84%

**Table 6:** Data-mining results by model of day segment and class.

Day Stage		Precision	Recall	F1 Score	MCC
Night 00:00–06:00	0	0.98	0.99	0.98	
	1	0.98	0.96	0.97	0.96
	2	0.98	0.98	0.98	
Morning 06:00–11:59	0	0.87	0.95	0.91	
	1	0.94	0.85	0.89	0.81
	2	0.88	0.80	0.84	
Afternoon 12:00–17:59	0	0.78	0.91	0.84	
	1	0.81	0.70	0.75	0.69
	2	0.87	0.72	0.79	
Evening 18:00–23:59	0	0.87	0.96	0.91	
	1	0.90	0.84	0.87	0.82
	2	0.94	0.83	0.88	
Note: 0 means healthy control, 1 depressive, and 2 schizophrenic episodes.					

## Data Availability

The dataset can be accessed via: http://datasets.simula.no/ accessed on 15 January 2022.
